# Congenital Heart Disease After Mid-Age: From the “Grown-Up” to the Elderly

**DOI:** 10.3390/diagnostics15040481

**Published:** 2025-02-17

**Authors:** Francesca Bonanni, Chiara Servoli, Gaia Spaziani, Elena Bennati, Chiara Di Filippo, Giulia Ksenia Cirri, Marzia Giaccardi, Iacopo Olivotto, Silvia Favilli

**Affiliations:** 1 ACHD Unit, Department of Cardiology, Meyer Children’s Hospital IRCCS, 50139 Florence, Italy; frabonanni95@gmail.com (F.B.); chiara.servoli@unifi.it (C.S.); gaia.spaziani@meyer.it (G.S.); elena.bennati@meyer.it (E.B.); cdf.cardio@gmail.com (C.D.F.); giuliaksenia@gmail.com (G.K.C.); iacopo.olivotto@meyer.it (I.O.); 2 Department of Cardiology, Santa Maria Annunziata Hospital, 50012 Florence, Italy; marzia.giaccardi@uslcentro.toscana.it

**Keywords:** adult congenital heart disease, elderly, frailty, comorbidities

## Abstract

Early surgery and improved medical care have led to the increased survival of neonates with congenital heart disease (CHD), who now commonly reach adulthood. Among adults with CHD, a growing subgroup is represented by middle-aged and even elderly patients. In this elderly population, acquired cardiac and extracardiac comorbidities represent the main cause of morbidity and mortality; the control and correction of cardiovascular risk factors or an appropriate check for extracardiac complications (such as malignancies) is therefore of paramount importance. Complications and frailty syndrome appear to occur earlier in ACHD than in the general population due to a frequent discrepancy between chronological and biological age. Multiple stressors throughout life (hemodynamic abnormalities, cardiac operations and interventional procedures, the placement of foreign materials) that result in a chronic inflammatory response are among the leading causes of premature senescence. This review is aimed at assessing the characteristics and special needs of this elderly ACHD population with a view to proposing novel models for the organization of extended care.

## 1. Introduction

Thanks to early surgery and improved medical care, today, the vast majority of newborns born with congenital heart disease (CHD), even complex ones, survive childhood and reach adulthood [1].

As underlined by the latest European guidelines [2], not only the number of adults suffering from CHD has exceeded that of children, but the average age is increasing, leading to the historical acronym “GUCH” (Grown-up Congenital Heart) being changed to ACHD (adult congenital heart disease). Furthermore, in recent decades, a small but significant subgroup of patients with ACHD over the age of 60 has come to represent a new “geriatric” population, increasingly discussed in the literature. In a large nationwide registry, the large majority (90%) of patients with mild CHD but also 75% of patients with moderate and 40% with complex CHD are reported to reach the age of 60 years [3]. CHDs are defined as “mild”, “moderate”, “severe” and “complex” according to the classification proposed by ESC guidelines [2]. It is estimated that 11% of the total ACHD population will reach or exceed the age of 60 years by 2030 [2].

Patients with ACHD are defined as “middle-age” and “elderly” when they are over 50 years old and over 65 years old, respectively.

The first clinical study specifically addressing elderly ACHD was published in 2011 by Afilalo et al. [4]. The authors established a consistent prevalence of ACHD (from 3.8 to 3.7 per 1000) in the older general population of Quebec over a 15-year period (1990–2005). In their cohort of elderly CHDs, the most frequent types of lesions were shunts (60%) followed by valvular lesions (37%), while “severe” CHD represented only 3%. Mortality was higher, as expected, in comparison with younger patients; survival was not predicted by the type of lesion and appeared to be related primarily to acquired medical conditions, in contrast with children, where mortality is related mainly to CHD severity, or with adolescents and adults, in whom the main determinants of death are ACHD-related complications (e.g., cyanosis, pulmonary hypertension, arrhythmias, etc.) or repeated cardiac surgery.

Dementia, chronic kidney disease and gastrointestinal bleeding (in patients taking anticoagulants or antiplatelet therapies) are frequently described in older patients with ACHD and adversely affect prognosis [4]. In general, among adults with CHD, the elderly population requires considerable resources and care, but dedicated expertise and resources are often lacking.

## 2. Characteristics of the Elderly ACHD Population

**Figure 1:**
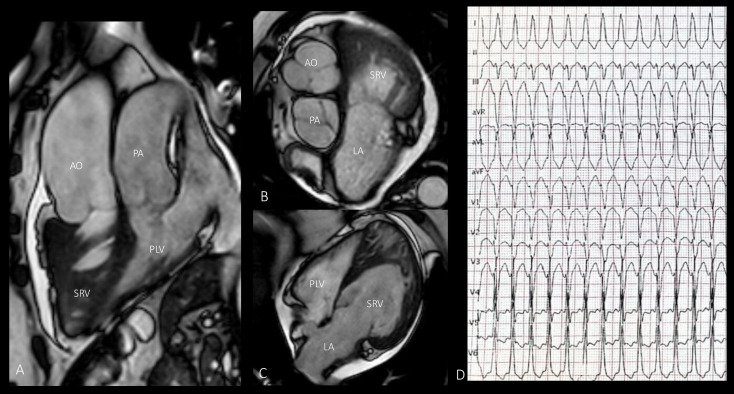
Diagnosis of congenitally corrected transposition of the great arteries (CCTGA) in a 69-year-old woman due to the onset of cardiac palpitations. (**A**) MRI shows a morphologically right systemic ventricle and a morphologically left subpulmonary ventricle. (**B**) The aorta is dilated and positioned anterior to and to the left of the pulmonary artery. The left atrium is also dilated. (**C**) Working against systemic resistance, the right ventricle has progressively enlarged and hypertrophied. (**D**) Electrocardiogram performed during symptoms shows supraventricular tachycardia with aberrancy. AO—aorta; LA—left atrium; PA—pulmonary artery; PLV—pulmonary left ventricle; SRV—systemic right ventricle.

Regarding composition, “simple” CHD in natural history and simple/moderately complex operated CHD represent the most common conditions in the elderly [3]. However, even patients with severe lesions may survive past mid-age, and occasionally, the first diagnosis may occur in their sixties, often because of arrhythmic complications (Figure 1).

Most operated patients should be considered “treated” but not “cured” [5]. Although survival beyond young adulthood with a good quality of life is reported in the majority, late complications are common and require highly specialized care [6]. The risk of developing atrial fibrillation and experiencing an ischemic stroke is much higher in adults with CHD < 65 years compared to the general population [7] and is expected to increase further in older patients.

Heart failure is common in ACHD and represents a leading cause of death [2]. Its incidence increases with aging, and underlying pathophysiology may vary in different conditions [8]. In a large Canadian ACHD cohort [9], the main predictors for the development of heart failure were age > 50 years, the severity of the congenital lesion, recent hospitalization for heart failure, pulmonary hypertension and the presence of coronary artery disease, obesity and systemic arterial hypertension [10].

Acquired heart disease represents a significant and growing concern in ACHD, especially in older patients. There are multiple reasons why adults with CHD have a greater risk of developing early atherosclerotic cardiovascular disease: the abnormal anatomy of coronary arteries, injuries during surgery (e.g., during “arterial switch” operation in transposition of the great arteries), aortic stiffness (e.g., in aortic coarctation), abnormal ventricular load conditions, chronic inflammation, etc. Chronic inflammation plays a role in the aging process because it increases, among other things, the risk of atherosclerosis and insulin resistance (hence the concept “inflamm-aging” proposed by Franceschi et al. [11]). Increased circulating cytokine levels have been reported in adults with CHD [12]. In the ACHD population, the role of inflammation, activated by multiple stressors throughout life, is highlighted by Brida et al. in a recent clinical consensus of the European Society of Cardiology [13].

## 3. New Therapeutic Challenges in the Management of Acquired Cardiovascular Risk Factors Among Adults with CHD

**Figure 2:**
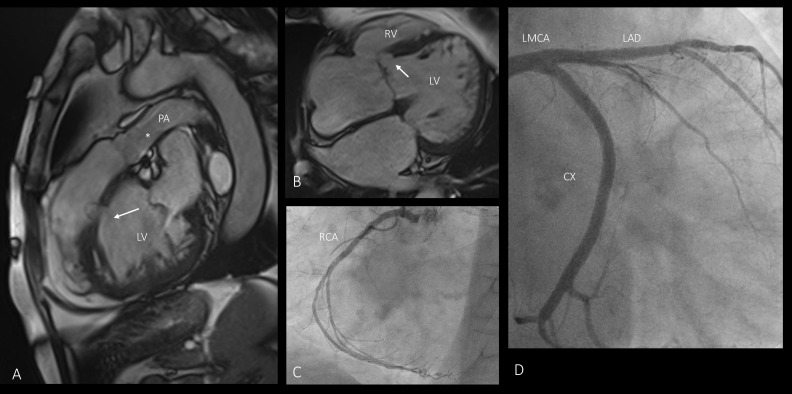
A 77-year-old male with a diagnosis of Double Inlet Left Ventricle (DILV) and Chronic Coronary Syndrome (CCS). He has a history of smoking, diabetes, stage II chronic kidney disease and hypothyroidism. He suffers from chronic atrial fibrillation, for which he has undergone numerous electrical cardioversions (ECVs) and is on AVK treatment. At the age of 57, he underwent coronary angioplasty and stent implantation on the left anterior descending artery (LAD). (**A**) MRI shows a large ventricular septal defect (➞) and hypoplasia of the pulmonary artery (*), and (**B**) a rudimentary right ventricle. (**C**) Angiography shows an irregular right coronary artery without critical stenosis. (**D**) The left main coronary artery is free from stenosis; the left anterior descending artery presents a good result from previous stenting in the proximal section; and the circumflex branch, codominant, is free from stenosis. CX—circumflex branch; LAD—left anterior descending artery; LMCA—left main coronary artery; LV—left ventricle; PA—pulmonary artery; RCA—right coronary artery; RV—right ventricle.

The improved survival of patients with CHD into adulthood has led to a significant shift in the focus of medical care, transitioning from childhood surgical repair to the long-term management of chronic conditions. As this population ages, they face an increasing burden of acquired cardiovascular risk factors and comorbidities, which not only contribute to premature cardiovascular morbidity but also complicate the management of underlying congenital defects. As a result, addressing and managing these acquired risk factors has become essential in the care of adults with CHD (Figure 2).

These issues highlight the critical importance of tailored management strategies incorporating cardiovascular risk assessment in the comprehensive care of these patients [4], particularly in individuals with congenital coronary artery anomalies, transposition of the great arteries (TGA) following arterial switch repair, Ross procedures, coarctation of the aorta, left ventricular outflow tract (LVOT) obstruction or external compression of the left coronary ostium, such as from a dilated pulmonary artery in Eisenmenger syndrome [2]. Furthermore, systemic or pulmonary hypertension may result in severe clinical manifestations, including angina, dyspnea or sudden cardiac death, necessitating heightened surveillance and prompt intervention.

Ischemia in ACHD may be challenging to suspect due to the frequent baseline abnormalities on electrocardiograms, such as prolonged QRS duration, bundle branch block and ventricular hypertrophy. These alterations require careful interpretation and underscore the value of patients having their baseline ECG readily accessible [13]. Given the inherent structural and surgical complexities and pre-existing hemodynamic burden, pharmacological and lifestyle interventions targeting modifiable cardiovascular risk factors should remain the primary focus in ACHD care.

### 3.1. Systemic Hypertension

Hypertension is one of the most commonly encountered comorbidities in ACHD, with a higher prevalence than that in the general population [4,14]. Its etiology is multifactorial, involving factors such as arterial stiffness, endothelial dysfunction, renal abnormalities, obstructive sleep apnea syndrome (OSAS) and specific congenital conditions like coarctation of the aorta or LVOT obstruction [13]. Elevated blood pressure is associated with adverse features, including left ventricular hypertrophy, accelerated aortic dilation, renal function deterioration and reduced functional capacity. It also contributes to the formation of vascular plaques, promoting coronary atherosclerosis [15]. When feasible, routine blood pressure measurements during clinic visits should be supplemented by home monitoring. In patients with a history of aortic coarctation repair, assessing blood pressure in both the right arm and lower extremities is critical for identifying any pressure gradients indicative of re-coarctation [16].

Current clinical guidelines emphasize the importance of strict blood pressure control to mitigate these risks. Therapeutic targets are often set lower than those recommended for the general population. Achieving a systolic blood pressure below 130 mmHg is a priority for most patients, while even more stringent targets may benefit those with significant aortic pathology [13,17]. The pharmacological management of hypertension in ACHD is tailored to the underlying congenital defect and its associated complications. Renin–angiotensin system inhibitors, such as angiotensin-converting enzyme inhibitors and angiotensin II receptor blockers, are frequently prescribed due to their dual effects in lowering blood pressure and reducing afterload. Beta-blockers are often employed for their hemodynamic advantages, particularly in patients with systemic right ventricles or those at risk for aortic dissection.

However, caution is warranted when using vasodilators in certain settings. In individuals with significant re-coarctation, these agents should be avoided when possible, as they can lead to pre-renal acute kidney injury. Similarly, in patients with Eisenmenger syndrome, vasodilators should be used sparingly because they may exacerbate right-to-left shunting, worsening systemic hypoxemia [2,13].

### 3.2. Obesity and Metabolic Syndrome

The increasing prevalence of obesity and metabolic syndrome among adults with ACHD represents a significant challenge in the landscape of lifelong cardiovascular management. Nearly half of patients with ACHD are overweight or obese, mirroring trends in the general population and emphasizing the pervasive impact of a sedentary lifestyle and environmental factors on this vulnerable cohort [18]. Notably, metabolic syndrome is more prevalent among patients with ACHD than in the general population, particularly as they age [19]. These conditions are closely tied to a constellation of risk factors, including systemic inflammation, arterial stiffness and endothelial dysfunction, which are often compounded by reduced physical activity, exercise restrictions and overprotective behaviors [19].

Obesity and metabolic syndrome have profound implications for patients with ACHD, exacerbating the cardiovascular workload and promoting insulin resistance and dyslipidemia, creating a vicious cycle of metabolic dysfunction, which heightens the risk of atherosclerotic cardiovascular diseases [18,20,21].

Addressing obesity and metabolic syndrome in ACHD requires a comprehensive approach that prioritizes preventive care. Routine screening and proper treatments for risk factors are essential. Nutritional counseling and behavioral interventions form the basis of treatment, emphasizing decreasing caloric intake and promoting a balanced diet [16]. Cardiac rehabilitation programs, in particular, have shown promise in improving this population’s metabolic parameters and functional capacity, underscoring the importance of a multidisciplinary approach to care [19,22]. However, treatment plans must account for the complexities of congenital heart anomalies and their long-term sequelae, ensuring that interventions do not exacerbate underlying pathophysiological conditions.

### 3.3. Physical Inactivity and Its Consequences

Physical inactivity remains a significant issue among patients with ACHD, primarily rooted in historical habits discouraging exercise due to concerns over exacerbating cardiovascular complications. However, current evidence underscores the adverse effects of sedentary behavior, including diminished peak oxygen uptake (VO_2_ max), heightened BMI and compromised mental health [22,23]. Tailored exercise programs, designed according to individual hemodynamic profiles, have emerged as integral components of modern ACHD management [24]

Aerobic activities like walking, swimming or cycling enhance cardiovascular fitness and improve metabolic markers like insulin sensitivity and lipid profiles. Structured exercise regimens have safely elevated exercise capacity, particularly when coupled with motivational techniques. Additionally, wearable fitness devices and telemedicine platforms are valuable tools for promoting adherence, offering real-time feedback and engaging patients [25,26].

The importance of physical activity counseling in routine ACHD care cannot be overstated, as it holds the potential to mitigate psychosocial burdens, including anxiety and depression, while fostering long-term health [13,22].

### 3.4. Dyslipidemia and Glucose Metabolism

Dyslipidemia is a well-established risk factor in ACHD, contributing to the early onset of atherosclerosis. Regular screening for lipid abnormalities is crucial, particularly in high-risk subgroups such as patients with a history of coarctation of the aorta or those who have undergone arterial switch repair for TGA or Ross surgery [13,17]. More stringent lipid control may be beneficial in these groups to mitigate cardiovascular risks. While statins are the first-line therapy for lowering LDL cholesterol, adding ezetimibe or PCSK9 inhibitors may be considered for patients who do not achieve target lipid levels [27]. Liver function should be monitored regularly in patients on statin therapy, especially in those with Fontan circulation or chronic right-sided heart failure, as these conditions may increase the risk of hepatic dysfunction [13,28]. Notably, statin prescription rates in patients with ACHD may be lower compared to the general population [28].

Lifestyle modifications, including dietary changes to reduce saturated fat intake, remain crucial for dyslipidemia management [29].

Impaired glucose metabolism, including insulin resistance and type 2 diabetes, is increasingly observed in patients with ACHD and is closely linked to metabolic syndrome [14]. Given its prolonged preclinical phase, screening with HbA1c measurements is critical. For frail patients, the target HbA1c should be set at <7% (53 mmol/mol). Metformin remains the first-line therapy, while GLP-1 receptor agonists and SGLT2 inhibitors are emerging as valuable options, particularly in patients with coexisting cardiovascular complications.

### 3.5. Integrated Approaches to Risk Factor Management

Managing acquired cardiovascular risk factors in ACHD requires an integrated, multidisciplinary approach. Collaboration is essential to develop individualized care plans that address the unique needs of this population [13]. Regular monitoring, patient education and engagement are key to effective long-term management.

Addressing anxiety and depression is also crucial, as many patients with ACHD face psychological challenges. Behavioral programs that build resilience and support lifestyle changes can improve quality of life and clinical outcomes [16,17]. Additionally, healthcare systems must adapt to the growing ACHD population by expanding access to specialized care and integrating preventive strategies into routine practice [17].

## 4. Biological Versus Chronological Age

In a recent state-of-the-heart review, Moons and Marelli address the discrepancy between chronological and biological age in ACHD (“young patients with old hearts”) [14]. The discrepancy is even more evident in patients with syndromic ACHD. This “accelerated aging” has important clinical and therapeutic implications. Not only do cardiovascular complications occur at younger ages compared with the general non-ACHD population, but also neurological decline and dementia are reported earlier in life.

The multiple stresses experienced by patients with CHD throughout their lives, linked to the congenital malformation but also to repeated cardiac surgery or interventional procedures and the implantation of foreign materials, and the resulting inflammatory response have been reported as causes of premature senescence both at the cardiac and systemic levels [30].

Regarding cardiovascular complications, atrial fibrillation (AF) increases with age in patients with ACHD, reaching a plateau around 70 years [31,32].

Compared with the general non-ACHD population, AF occurs approximately 30 years earlier and the risk is 20- and 10-fold higher in the decades 50–54 and 55–59, respectively. It is not surprising that the risk of developing AF is greater in patients with complex CHD with broader systemic repercussions, such as Ebstein anomaly.

AF increases morbidity and mortality in patients with ACHD, and the risk of developing ischemic stroke is greater even in those with a low CHA_2_DS_2_-VASc score (0–1) [33,34]. As for other chronic heart diseases of the young, such as cardiomyopathies [35], thromboembolic risk stratification based on a traditional risk score is not suitable for patients with ACHD, who are often younger compared to the general population considered for anticoagulation.

Among extracardiac complications, an increased risk of cancer at younger ages has been reported in patients with ACHD, representing a relevant cause of premature mortality [36,37].

There are multiple mechanisms hypothesized to explain the higher rate of malignancies in the ACHD population, from increased radiation exposure resulting from diagnostic or interventional procedures, especially when performed during childhood, to genetic mutations and inappropriate lifestyles (particularly, limited physical activity).

An increased risk of malignancies has been described in cyanotic CHD due to chronic hypoxia and in patients with Fontan circulation. In particular, cavopulmonary connection Hepatocellular Carcinoma, secondary elevated central venous pressures and liver fibrosis, and neuroendocrine tumors (probably related to chronic hypoxia) are the most commonly described [37].

Genetic predisposition to tumors is well known in syndromes frequently associated with CHD, from Down syndrome, in which patients present a greater risk of developing leukemia, independently of CHD, to DiGeorge syndrome or rasopathies. Furthermore, the presence of damaging gene variants which might contribute to both CHD and cancer was discussed in a recent study [36].

Several authors report an increased risk of neurological damage and dementia in ACHD. The multiple factors which can lead to brain injury and neurodevelopmental disorders in these patients, starting from the first years of life, are discussed by Marelli and co. in a very interesting paper exploring the close relationship between cardiovascular and neurovascular disease as patients with ACHD age. Genetic mutations that affect brain development, altered brain perfusion often early in fetal life, repeated interventions and, later in life, the previously discussed acquired cardiovascular comorbidities (hypertension, diabetes, coronary artery disease) can cumulate to determine brain injury, cognitive decline and premature dementia [38].

Awareness of these new healthcare needs should lead to careful follow-up, which cannot be limited to cardiological evaluation, and to a new organization of medical and social support for the “elderly” ACHD population [39].

## 5. Frailty in CHD After Mid-Age

Awareness of age-related extracardiac and cardiac comorbidities and of the earlier aging of patients with ACHD has led to investigation of the issue of frailty, which is well assessed in the elderly general population and likely manifests prematurely in adults with CHD.

Fried’s definition of the frailty phenotype is based on five criteria: weakness, slow walking, involuntary weight loss, decreased physical activity, and fatigue [40]. There is growing evidence that cognitive impairment and frailty are related to a worse prognosis and should guide clinical and therapeutic choices.

Although studies on this subject are still limited, frailty in patients with ACHD has been reported more often in low-income countries, suggesting a role of socioeconomic factors [41,42].

Recently, in a large multicenter study including 814 patients with ACHD with a median age of 52 years, a frailty phenotype was found in 5.8%, while 41.9% were defined as “pre-frail” [41,43]. Overall, nearly half of the study population was classified as frail or pre-frail; the same percentage is reported in the general population aged 65 years or older, confirming the concept of “earlier aging” for patients with ACHD. Moreover, nearly 40% of patients presented some degree of neurologic impairment. Risk factors for frailty were represented by older age, female sex, the presence of comorbidities and a higher physiologic class (as defined by American ACHD guidelines). In addition to the previous risk factors, pre-frailty was associated with milder defects.

Sarcopenia is an important component of frailty syndrome. The risk of sarcopenia and cachexia is substantial in elderly adults with CHD due to the possible development of heart failure secondary to CHD. Nutritional advice and, if necessary, specific macro- and micronutrient supplementation should be taken into consideration [44]. On the other hand, progressive muscle wasting is largely described in severe CHD, such as Fontan circulation [45,46], suggesting that nutritional problems should be considered earlier in order to limit premature exercise impairment.

## 6. Conclusions

The progressive increase in “elderly” patients with ACHD, who present acquired cardiac and extracardiac comorbidities as well as long-term complications related to their congenital malformation, requires a change in programs of care.

In older patients, together with control of CHD-related sequelae and residual defects, a priority is represented by the strict control and prompt correction of cardiovascular risk factors (diabetes, hypertension, obesity, low physical activity) in order to prevent atherosclerosis-related complications. The concept of the “early aging” of patients with ACHD should be taken into account to guide follow-up and therapies.

The complex interplay between congenital abnormalities and acquired comorbidities enhances frailty in these patients, which often occurs at an earlier age in comparison to the general population.

The development of preventive care programs, which should begin in early adulthood, requires dedicated resources and inevitably results in additional economic costs. However, these costs should be balanced with a possible decrease in admissions to emergency departments and general hospitals, as well as socioeconomic costs related to premature aging and the frailty of adults with CHD.

Targeted preventive strategies should likely be developed in different ACHD subpopulations.

Dedicated studies will better define the therapeutic challenges and special needs of this growing subgroup of the ACHD population.

## References

[1] Marelli A.J., Ionescu-Ittu R., Mackie A.S., Guo L., Dendukuri N., Kaouache M. (2014). Lifetime prevalence of congenital heart disease in the general population from 2000 to 2010. Circulation.

[2] Baumgartner H., De Backer J., Babu-Narayan S.V., Budts W., Chessa M., Diller G.P., Lung B., Kluin J., Lang I.M., Meijboom F. (2021). 2020 ESC Guidelines for the management of adult congenital heart disease. Eur. Heart J..

[3] van der Bom T., Mulder B.J., Meijboom F.J., van Dijk A.P., Pieper P.G., Vliegen H.W., Konings T.C., Zwinderman A.H., Bouma B.J. (2015). Contemporary survival of adults with congenital heart disease. Heart.

[4] Afilalo J., Therrien J., Pilote L., Ionescu-Ittu R., Martucci G., Marelli A.J. (2011). Geriatric Congenital Heart Disease: Burden of Disease and Predictors of Mortality. J. Am. Coll. Cardiol..

[5] Mandalenakis Z., Skoglund K., Dellborg M. (2019). Congenital heart disease: The children will become elderly. Aging.

[6] Baumgartner H. (2014). Geriatric congenital heart disease: A new challenge in the care of adults with congenital heart disease?. Eur. Heart J..

[7] Holmgren A., Giang K.W., Fedchenko M., Eriksson P., Dellborg M., Mandalenakis Z. (2024). Ischemic Stroke in Patients with Congenital Heart Disease and Atrial Fibrillation. J. Am. Heart Assoc..

[8] Opotowsky A.R. (2024). The Pathophysiology(ies) of Heart Failure in Adults with Congenital Heart Disease. Heart Fail. Clin..

[9] Watelle L., Roy L.-O., Lauzon-Schnitka J., Newell G., Dumas A., Nadeau A., Xiong W.T., Rego K., Beaulieu C., Groulx-Boivin E. (2023). The Quebec Congenital Heart Disease Registry: A Model of Prospective Databank to Facilitate Research in Congenital Cardiology. CJC Pediatr. Congenit. Hear. Dis..

[10] Cohen S., Liu A., Wang F., Guo L., Brophy J.M., Abrahamowicz M., Therrien J., Beauchesne L.M., Bédard E., Grewal J. (2021). Risk prediction models for heart failure admissions in adults with congenital heart disease. Int. J. Cardiol..

[11] Franceschi C., Bonafe M., Valensin S., Olivieri F., De Luca M., Ottaviani E., De Benedictis G. (2000). Inflamm-aging: An evolutionary perspective on immunosenescence. Ann. N. Y. Acad. Sci..

[12] Sharma R., Bolger A.P., Li W., Davlouros P.A., Volk H.-D., Poole-Wilson P.A., Coats A.J., Gatzoulis M.A., Anker S.D. (2003). Elevated circulating levels of inflammatory cytokines and bacterial endotoxin in adults with congenital heart disease. Am. J. Cardiol..

[13] Brida M., De Rosa S., Legendre A., Ladouceur M., Dos Subira L., Scognamiglio G., Di Mario C., Roos-Hesselink J., Goossens E., Diller G. (2023). Acquired cardiovascular disease in adults with congenital heart disease A call to action for timely preventive measures—A clinical consensus statement of the European Society of Cardiology Working Group on Adult Congenital Heart Disease in collaboration with the European Association of Preventive Cardiology and the European Association of Percutaneous Cardiovascular Interventions. Eur. Heart J..

[14] Moons P., Marelli A. (2022). Born to Age: When Adult Congenital Heart Disease Converges with Geroscience. JACC Adv..

[15] McEvoy J.W., McCarthy C.P., Bruno R.M., Brouwers S., Canavan M.D., Ceconi C., Christodorescu R.M., Daskalopoulou S.S., Ferro C.J., Gerdts E. (2024). 2024 ESC Guidelines for the management of elevated blood pressure and hypertension. Eur. Heart J..

[16] Stout K.K., Daniels C.J., Aboulhosn J.A., Bozkurt B., Broberg C.S., Colman J.M., Crumb S.R., Dearani J.A., Fuller S., Gurvitz M. (2019). 2018 AHA/ACC Guideline for the Management of Adults with Congenital Heart Disease: A Report of the American College of Cardiology/American Heart Association Task Force on Clinical Practice Guidelines. J. Am. Coll. Cardiol..

[17] Tutarel O. (2014). Acquired heart conditions in adults with congenital heart disease: A growing problem. Heart.

[18] Willinger L., Brudy L., Meyer M., Oberhoffer-Fritz R., Ewert P., Müller J. (2021). Overweight and obesity in patients with congenital heart disease: A systematic review. Int. J. Environ. Res. Public Health.

[19] Niwa K. (2021). Metabolic syndrome and coronary artery disease in adults with congenital heart disease. Cardiovasc. Diagn. Ther..

[20] Mottillo S., Filion K.B., Genest J., Joseph L., Pilote L., Poirier P., Rinfret S., Schiffrin E.L., Eisenberg M.J. (2010). The Metabolic Syndrome and Cardiovascular Risk: A Systematic Review and Meta-Analysis. J. Am. Coll. Cardiol..

[21] Deen J.F., Krieger E.V., Slee A.E., Arslan A., Arterburn D., Stout K.K., Portman M.A. (2016). Metabolic Syndrome in Adults with Congenital Heart Disease. J. Am. Heart Assoc. Cardiovasc. Cerebrovasc. Dis..

[22] Longmuir P.E., Brothers J.A., De Ferranti S.D., Hayman L.L., Van Hare G.F., Matherne G.P., Davis C.K., Joy E.A., McCrindle B.W., American Heart Association Atherosclerosis, Hypertension and Obesity in Youth Committee of the Council on Cardiovascular Disease in the Young (2013). Promotion of physical activity for children and adults with congenital heart disease: A scientific statement from the American Heart Association. Circulation.

[23] Kempny A., Dimopoulos K., Uebing A., Moceri P., Swan L., Gatzoulis M.A., Diller G.-P. (2012). Reference values for exercise limitations among adults with congenital heart disease. Relation to activities of daily lifesingle centre experience and review of published data. Eur. Heart J..

[24] Budts W., Börjesson M., Chessa M., van Buuren F., Trindade P.T., Corrado D., Heidbuchel H., Webb G., Holm J., Papadakis M. (2013). Physical activity in adolescents and adults with congenital heart defects: Individualized exercise prescription. Eur. Heart J..

[25] Pelliccia A., Sharma S., Gati S., Bäck M., Börjesson M., Caselli S., Collet J.-P., Corrado D., Drezner J.A., Halle M. (2021). 2020 ESC Guidelines on sports cardiology and exercise in patients with cardiovascular disease. Eur. Heart J..

[26] Borrelli N., Grimaldi N., Papaccioli G., Fusco F., Palma M., Sarubbi B. (2023). Telemedicine in Adult Congenital Heart Disease: Usefulness of Digital Health Technology in the Assistance of Critical Patients. Int. J. Environ. Res. Public Health.

[27] Marx N., Federici M., Schütt K., Müller-Wieland D., A Ajjan R., Antunes M.J., Christodorescu R.M., Crawford C., Di Angelantonio E., Eliasson B. (2023). 2023 ESC Guidelines for the management of cardiovascular disease in patients with diabetes: Developed by the task force on the management of cardiovascular disease in patients with diabetes of the European Society of Cardiology (ESC). Eur. Heart J..

[28] Flannery L.D., Fahed A.C., Yeh D.D., Youniss M.A., Barinsky G.L., Schmidt A.C.S., Benavidez O.J., Meigs J.B., Bhatt A.B. (2018). Frequency of Guideline-Based Statin Therapy in Adults with Congenital Heart Disease. Am. J. Cardiol..

[29] Mach F., Baigent C., Catapano A.L., Koskinas K.C., Casula M., Badimon L., Chapman M.J., De Backer G.G., Delgado V., Ference B.A. (2020). 2019 ESC/EAS Guidelines for the management of dyslipidaemias: Lipid modification to reduce cardiovascular risk: The Task Force for the management of dyslipidaemias of the European Society of Cardiology (ESC) and European Atherosclerosis Society (EAS). Eur. Heart J..

[30] Iacobazzi D., Alvino V.V., Caputo M., Madeddu P. (2022). Accelerated Cardiac Aging in Patients with Congenital Heart Disease. Front. Cardiovasc. Med..

[31] Ebrahim M.A., Escudero C.A., Kantoch M.J., Vondermuhll I.F., Atallah J. (2018). Insights on Atrial Fibrillation in Congenital Heart Disease. Can. J. Cardiol..

[32] Wu M.H., Chiu S.N., Tseng W.C., Lu C.W., Kao F.Y., Huang S.K. (2023). Atrial fibrillation in adult congenital heart disease and the general population. Heart Rhythm..

[33] Kartas A., Papazoglou A.S., Moysidis D.V., Despotopoulos S., Baroutidou A., Kosmidis D., Koutsakis A., Liori S., Apostolopoulou S., Frogoudaki A. (2024). Use of apixaban in adults with congenital heart disease and atrial arrhythmias: The PROTECT-AR study. Int. J. Cardiol..

[34] Lin Y.-S., Huang Y.-C., Lin C.-P., Wu V.C.-C., Kao Y.-W., Chiang H.-Y., Chu P.-H. (2023). Atrial Fibrillation in Adult Congenital Heart Increase Ischemic Stroke Risk Even at Low CHA2DS2-VASc Score. Rev. Cardiovasc. Med..

[35] Arbelo E., Protonotarios A., Gimeno J.R., Arbustini E., Barriales-Villa R., Basso C., Bezzina C.R., Biagini E., A Blom N., A de Boer R. (2023). 2023 ESC Guidelines for the management of cardiomyopathies: Developed by the task force on the management of cardiomyopathies of the European Society of Cardiology (ESC). Eur. Heart J..

[36] Morton S.U., Shimamura A., Newburger P.E., Opotowsky A.R., Quiat D., Pereira A.C., Jin S.C., Gurvitz M., Brueckner M., Chung W.K. (2021). Association of Damaging Variants in Genes with Increased Cancer Risk Among Patients with Congenital Heart Disease. JAMA Cardiol..

[37] Lakshmanan S., Gimelli A. (2023). Cancer risk in adult congenital heart disease. Int. J. Cardiol. Congenit. Heart Dis..

[38] Marelli A., Miller S.P., Marino B.S., Jefferson A.L., Newburger J.W. (2016). Brain in Congenital Heart Disease Across the Lifespan: The Cumulative Burden of Injury. Circulation.

[39] Tournoy T.K., Moons P., Daelman B., De Backer J. (2023). Biological Age in Congenital Heart Disease—Exploring the Ticking Clock. J. Cardiovasc. Dev. Dis..

[40] Fried L.P., Tangen C.M., Walston J., Newman A.B., Hirsch C., Gottdiener J., Seeman T., Tracy R., Kop W.J., Burke G. (2001). Frailty in Older Adults: Evidence for a Phenotype. J. Gerontol. Ser. A.

[41] Daelman B., Van Bulck L., Luyckx K., Kovacs A.H., Van De Bruaene A., Ladouceur M., Yang H.-L., Moon J.R., Schmidt A., Lykkeberg B. (2024). Frailty and Cognitive Function in Middle-Aged and Older Adults with Congenital Heart Disease. J. Am. Coll. Cardiol..

[42] Siriwardhana D.D., Hardoon S., Rait G., Weerasinghe M.C., Walters K.R. (2018). Prevalence of frailty and prefrailty among community-dwelling older adults in low-income and middle-income countries: A systematic review and meta-analysis. BMJ Open.

[43] Van Bulck L., Kovacs A.H., Goossens E., Luyckx K., Zaidi A., Wang J.-K., Yadeta D., Windram J., Van De Bruaene A., Thomet C. (2022). Rationale, design and methodology of APPROACH-IS II: International study of patient-reported outcomes and frailty phenotyping in adults with congenital heart disease. Int. J. Cardiol..

[44] Lorente M., Azpiroz M.J., Guedes P., Burgos R., Lluch A., Dos L. (2023). Nutrition, dietary recommendations, and supplements for patients with congenital heart disease. Int. J. Cardiol. Congenit. Heart Dis..

[45] Rychik J., Atz A.M., Celermajer D.S., Deal B.J., Gatzoulis M.A., Gewillig M.H., Hsia T.-Y., Hsu D.T., Kovacs A.H., McCrindle B.W. (2019). Evaluation and Management of the Child and Adult with Fontan Circulation: A Scientific Statement from the American Heart Association. Circulation.

[46] Tran D., D’Ambrosio P., Verrall C.E., Attard C., Briody J., D’Souza M., Singh M.F., Ayer J., D’Udekem Y., Twigg S. (2020). Body composition in young adults living with a fontan circulation: The myopenic profile. J. Am. Heart Assoc..

